# Positive Effects of Nonnative Invasive *Phragmites australis* on Larval Bullfrogs

**DOI:** 10.1371/journal.pone.0044420

**Published:** 2012-08-30

**Authors:** Mary Alta Rogalski, David Kiernan Skelly

**Affiliations:** School of Forestry and Environmental Studies, Yale University, New Haven, Conneticut, United States of America; Roehampton University, United Kingdom

## Abstract

**Background:**

Nonnative *Phragmites australis* (common reed) is one of the most intensively researched and managed invasive plant species in the United States, yet as with many invasive species, our ability to predict, control or understand the consequences of invasions is limited. Rapid spread of dense *Phragmites* monocultures has prompted efforts to limit its expansion and remove existing stands. Motivation for large-scale *Phragmites* eradication programs includes purported negative impacts on native wildlife, a view based primarily on observational results. We took an experimental approach to test this assumption, estimating the effects of nonnative *Phragmites australis* on a native amphibian.

**Methodology/Principal Findings:**

Concurrent common garden and reciprocal transplant field experiments revealed consistently strong positive influences of *Phragmites* on *Rana catesbeiana* (North American bullfrog) larval performance. Decomposing *Phragmites* litter appears to contribute to the effect.

**Conclusions/Significance:**

Positive effects of *Phragmites* merit further research, particularly in regions where both *Phragmites* and *R. catesbeiana* are invasive. More broadly, the findings of this study reinforce the importance of experimental evaluations of the effects of biological invasion to make informed conservation and restoration decisions.

## Introduction

Global travel and trade continue to introduce species to new environments at an unprecedented scale [1], challenging efforts to meet conservation goals. In the US alone, an estimated $25 billion are spent each year in management of invasive species [2] which are often presumed to have negative effects on native species [3]. The magnitude of the issue means that each effort directed at the control of invasive species must be allocated wisely. Unfortunately, our understanding of the effects invasive species have on native species, populations, communities and ecosystems is often fragmented [4]. A rising tide of evidence calls into question the nearly ubiquitous presumption that nonnative invasive species have negative effects on native taxa, with instead a mixture of positive, negative and neutral impacts observed [3].

*Phragmites australis* (Cav.) Trin. Ex Steud. (common reed) is one of the most well known and aggressively managed invasive plant species in the United States. While this robust perennial grass has inhabited North America for at least the past 40 000 years [5], an invasive, nonnative strain thought to originate from Eurasia most likely established in the northeastern US in the 19^th^ century [6]. Over the past several decades *Phragmites* has spread dramatically, especially in coastal regions of the northeastern and mid-Atlantic states, replacing millions of hectares of native plants in tidal wetlands alone [7]. Since the discovery of the invasive strain, a comprehensive quantitative assessment of the distribution of nonnative *Phragmites* has not been completed; however regional assessments made in several Atlantic states indicate that native *Phragmites* stands are rare [8].

Nonnative *Phragmites* forms dense monocultures with above ground biomass ranging from 1–3 kg/m^2^ [9]; biomass accumulation increases as an invasion matures [10]. *Phragmites* stands have a substantial, tight matrix of below ground roots and rhizomes, with lateral runners serving as an effective method of expansion. Ecosystem changes in hydrology, nutrient cycling and biomass accumulation are associated with *Phragmites* invasion [11].

Nonnative invasive plants are generally thought to support fewer wildlife species while providing habitat and food resources which may be inferior compared with those provided by native species that coevolved with the native fauna. Thus nonnative plants are perceived as a threat to native wildlife.

Amphibian population declines, which have been occurring globally over at least the past forty years, are in part attributed to negative impacts of invasive species [12]–[13]. While impacts of invasive pathogens, predators and competitors on amphibian populations have been thoroughly examined, just a handful of published studies have measured the effects of a nonnative plant on native larval amphibians [14]–[18].

In this study we assess the effects of nonnative *Phragmites* on a native amphibian, *Rana catesbeiana* (North American bullfrog). We focus on larval performance, a developmental period when *R. catesbeiana* might be expected to be most directly influenced by the effects of a nonnative emergent wetland plant. With over 100 live culms per m^2^ and with plants reaching heights of up to 4 m [9], *Phragmites* invasion in ponds could lead to colder water temperatures, which may slow larval development. Additionally, the thick layer of leaf detritus that accumulates in the littoral edge of *Phragmites* stands might support a different biofilm than that of native detritus. In general, leaf detritus of differing sources is known to yield very different decompositional patterns and biofilms in aquatic systems [19]; while nonnative plant invasion is capable of causing significant shifts in organic matter dynamics in wetlands [20]. Detritus and the associated biofilm constitute the chief portion of the diet of larval amphibians [21]–[22]. Interspecific differences in litter quality such as lability and C:N ratio can affect leaf breakdown [19], and may influence amphibian larval development [16], [18]. Thus a major shift in available detritus could have strong impacts on amphibian larval performance.

We take an experimental approach to evaluate the influence of *Phragmites* on *R. catesbeiana* larval performance (growth, development and survival). Questions of interest include 1) How does the presence of nonnative *Phragmites* influence native amphibian larval performance? 2) More specifically, how does larval performance differ when provided a diet composed of *Phragmites* detritus compared with alternative native detritus? 3) If *Phragmites* influences amphibian larval performance, have amphibian populations diverged in response to the effects?

We used a field reciprocal transplant experiment to estimate the overall effect of *Phragmites* on *R. catesbeiana* larvae. A concurrent common garden experiment was used to evaluate how *Phragmites* detritus affected larval performance absent other related changes in the natural environment. The strategy of using two complementary experimental venues was chosen to strengthen the basis of support for any discovered patterns. Both experiments were designed to reveal evidence of divergence among populations either exposed or naïve to the influence of *Phragmites*. Contemporary evolution, evolution occurring in ecologically relevant time scales, has been found to be much more common than was previously appreciated [23], even in organisms with relatively longer generation times such as amphibians [24]–[25]. Anthropogenic environmental changes, including introduced species, appear to influence stronger phenotypic responses in wild animal populations than more ‘natural’ changes [26]. Thus, if strong effects of *Phragmites* were discovered, it would not be unreasonable to find that *R. catesbeiana* populations have adapted to these effects.

## Results

**Figure 1:**
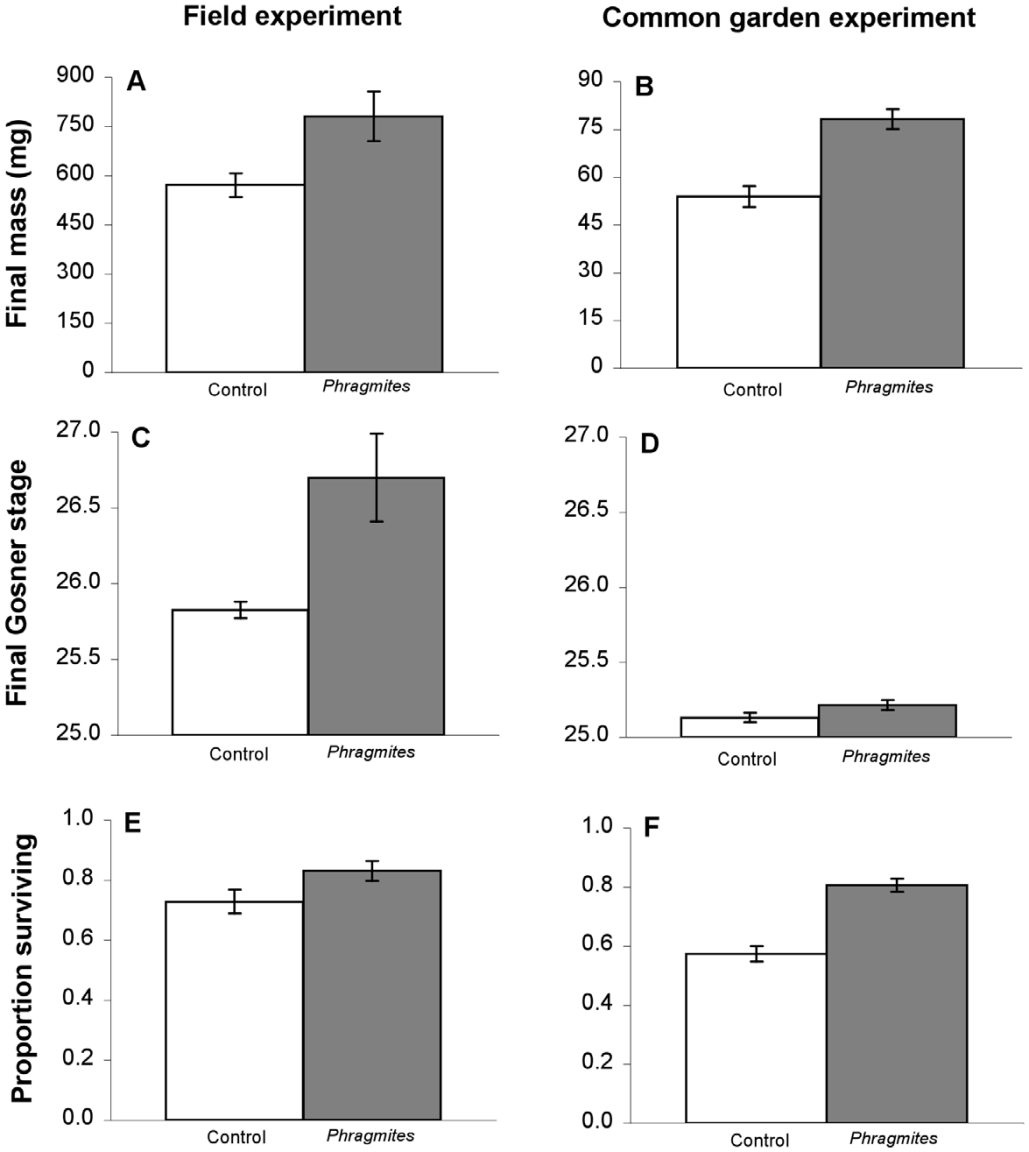
The effects of *Phragmites australis* on amphibian larval growth and development. Untransformed data from reciprocal transplant and common garden experiments including final mass (mg) (a, b), Gosner developmental stage (c, d) and proportion surviving (e, f) after six weeks of growth. Bars represent ±1 standard error. Dark columns represent *Phragmites* treatment, light is control. Note differing axis scales for final mass in Fig. 1a and b. * Represents a statistically significant difference (p value <0.05) between the two treatments found in the ANOVA test.

The common garden and reciprocal transplant field experiments revealed a consistently strong positive influence of *Phragmites* on *R. catesbeiana* larval performance (Table 1, Table 2, Figure 1). Multivariate and univariate analyses of variance yielded differences according to treatment (*Phragmites* vs. control) for every amphibian performance measure in each experiment (with the exception of developmental stage in the common garden setting, where the effect was marginally significant). Mass and survival rate showed large differences among treatment groups. Tadpoles in the *Phragmites* treatments weighed 37% more and survived 34% better in the common garden experiment and were 31% heavier and survived 13% better in the field experiment (Figure 1). In the field experiment, developmental stage of tadpoles raised in *Phragmites* wetlands was relatively more advanced than those raised in non-*Phragmites* wetlands. In all cases, origin (natal pond type) and the interaction between origin and treatment were not significant.

**Table 1:** Multivariate and univariate Analyses of Variance results for the field experiment (n = 68 enclosures) showing effects of origin (*Phragmites* or control), vegetation treatment (*Phragmites* or native deciduous leaf litter control), and the interaction between origin and treatment on the growth (final mass), survival rate, and final developmental stage of larval *Rana catesbeiana*.

Field Experiment Variable and fixed effects	df	F	P
MANOVA model (Wilk’s Lambda)	3, 62		
Origin	1	0.84	0.4784
Treatment	1	4.78	0.0046
Origin×Treatment	1	1.06	0.3718
Log Mass (mg)	3, 64		
Origin	1	0.01	0.9376
Treatment	1	5.07	0.0277
Origin×Treatment	1	0.50	0.4841
ArcSine Square Root Survival	3, 64		
Origin	1	0.61	0.4375
Treatment	1	5.39	0.0235
Origin×Treatment	1	1.80	0.1849
Log Stage	3, 64		
Origin	1	0.85	0.3589
Treatment	1	8.94	0.0040
Origin×Treatment	1	1.02	0.3173

**Table 2:** Multivariate and univariate Analyses of Variance results for the common garden experiment (n = 80 containers) showing effects of origin (*Phragmites* or control), vegetation treatment (*Phragmites* or native deciduous leaf litter control), and the interaction between origin and treatment on the growth (final mass), survival rate, and final developmental stage of larval *Rana catesbeiana*.

Common Garden Experiment Variable and fixed effects	df	F	*p*
MANOVA model (Wilk’s Lambda)	3, 74		
Origin	1	1.83	0.1489
Treatment	1	65.43	<0.0001
Origin×Treatment	1	1.24	0.3013
Log Mass (mg)	3, 76		
Origin	1	0.71	0.4028
Treatment	1	46.07	<0.0001
Origin×Treatment	1	0.22	0.6397
ArcSine Square Root Survival	3, 76		
Origin	1	0.66	0.4180
Treatment	1	43.81	<0.0001
Origin×Treatment	1	1.15	0.2868
Log Stage	3, 76		
Origin	1	0.90	0.3470
Treatment	1	3.79	0.0552
Origin×Treatment	1	0.41	0.5229

Analysis of water chemistry in the common garden experiment showed that dissolved oxygen was slightly higher in the *Phragmites* treatment (Table 3; control: 8.51 mg/L, SE 0.12; *Phragmites* 8.99 mg/L, SE 0.10). Temperature and pH did not differ between detritus types.

**Table 3:** Multivariate and univariate Analyses of Variance results showing effects of vegetation treatment (*Phragmites* or native leaf litter control) on: dissolved oxygen, pH and temperature in the common garden experiment; and dissolved oxygen, conductivity, pH and temperature in the field. a.

Variable and fixed effects	df	F	*p*	n
**Common Garden**				219 container days
MANOVA model (Wilk’s Lambda)	3, 215	3.37	0.0195	
Dissolved Oxygen	1, 217	9.14	0.0028	
pH	1, 217	3.66	0.0570	
Temperature	1, 217	0.21	0.6491	
**Field**				50 pond days
MANOVA model (Wilk’s Lambda)	3, 46	7.38	0.0004	
Dissolved Oxygen	1, 48	12.26	0.0010	
pH	1, 48	1.26	0.2668	
Conductivity	1, 48	4.35	0.0424	
Temperature a	1, 66	18.64	<0.0001	68 enclosures

a Temperature was not included in the MANOVA in the field experiment for reasons explained in the methods.

In the field experiment, dissolved oxygen was lower in *Phragmites* wetlands, with an average value of 3.77 mg/L (SE 0.50) compared with 6.07 mg/L (SE 0.42) in control wetlands (Table 3). Conductivity was higher in *Phragmites* wetlands (191.6 µS, SE 10.1 in *Phragmites*; 160.0 µS, SE 11.3 in control). pH did not differ according to treatment. The temperature experienced by animals in the field experiment was on average 1.48°C colder in *Phragmites* than in the control wetlands (mean temperature in non-*Phragmites* wetlands 26.72°C, SE 0.19; mean temperature in *Phragmites* wetlands 25.24°C, SE 0.21; n = 68 enclosures).

Characterization of detritus in the control wetlands showed *Quercus* Lobatae spp.(red oaks), *Fagus grandifolia* (beech) and *Acer rubrum* (red maple) to dominate in three wetlands, while *Typha latifolia* dominated one control wetland (Table 4). *Phragmites* wetland detritus consisted of 94–100% *Phragmites australis* leaf litter, with a small contribution of *Quercus* Lobatae spp, *A. rubrum*, or *Populus grandidentata* in some cases. One *Phragmites* wetland used in the study was found to have been chemically treated between the time of the study and the collection of the leaf litter for analysis, thus no litter species data was available for this wetland. However, red oak, red maple and cherry trees were growing near the *Phragmites* beds and may have contributed a small fraction (less than 5%) of the litter used in the reciprocal transplant. Additionally, one *Phragmites* wetland was used for litter in the common garden experiment but the data from this pond and its pair were excluded from the analysis of the reciprocal transplant or common garden experiments since only a single egg mass was found in this wetland.

**Table 4:** Characterization of leaf litter in control and *Phragmites* wetlands. a b c.

Pond	Treatment	Species	Common name	Proportion
C-1 a	Control	*Quercus* Lobatae spp.	Red oak spp.	0.46
		*Fagus grandifolia*	American beech	0.27
		*Acer rubrum*	Red maple	0.22
		*Quercus* Quercus spp.	White oak spp.	0.02
		*Betula lenta*	Black birch	0.01
		*Prunus serotina*	Black cherry	0.01
		*Salix* sp.	Willow sp.	0.01
				
C-2	Control	*Typha latifolia*	Broad-leaved cattail	1.0
				
C-3	Control	*Acer rubrum*	Red maple	0.56
		*Quercus* Lobatae spp.	Red oaks spp.	0.21
		*Betula lenta*	Black birch	0.08
		*Acer saccharum*	Sugar maple	0.07
		*Quercus* Quercus spp.	White oak spp.	0.06
		*Fagus grandifolia*	American beech	0.01
		*Prunus pensylvanica*	Pin cherry	0.01
				
C-4 a	Control	*Quercus* Lobatae spp.	Red oak spp.	0.69
		*Quercus* Quercus spp.	White oak spp.	0.16
		*Acer rubrum*	Red maple	0.13
		*Cornus* sp.	Dogwood sp.	0.01
				
P-1	*Phragmites*	*Phragmites australis*	Common reed	0.97
		*Quercus* Lobatae spp.	Red oaks	0.02
		*Acer rubrum*	Red maple	0.01
				
P-2	*Phragmites*	*Phragmites australis*	Common reed	0.94
		*Populus grandidentata*	Bigtooth aspen	0.03
		*Acer rubrum*	Red maple	0.01
				
P-3 b	*Phragmites*	*Phragmites australis*	Common reed	
				
P-4 a	*Phragmites*	*Phragmites australis*	Common reed	1.00
				
P-5 a c	*Phragmites*	*Phragmites australis*	Common reed	1.00

a Litter was collected from these wetlands for use in the common garden experiment.

b No litter was available during collection in this wetland. *Phragmites* likely contributed at least 95% of the litter with deciduous tree litter making up the remaining 5%.

c Litter contributed to *Phragmites* treatment in the common garden experiment. Data from this wetland and its pair were not analyzed in the common garden or reciprocal transplant experiments.

## Discussion

Nonnative *Phragmites* is widely seen as a noxious weed or even a villain [27]–[28] as it rapidly spreads throughout wetlands in the US, particularly along the northern and mid-Atlantic coast. Among several oft cited threats associated with *Phragmites*, negative effects on wildlife populations are prominent [29]–[33]. We evaluated this largely untested contention using experiments on native *R. catesbeiana*. Surprisingly, we discovered strong positive influences of *Phragmites* on *R. catesbeiana* larval performance.

Our finding is not likely to be an artifact of an experimental setup [34]. By performing complementary experiments in multiple venues, we attempted to reduce the likelihood of a misleading inference. In fact, we discovered strong concordance between results obtained in field enclosures designed to maximize the exposure of experimental animals to conditions associated with *Phragmites* dominated and *Phragmites* absent environments and a targeted common garden experiment conducted in artificial containers. In both contexts we observed sizable *Phragmites* associated increases in both tadpole growth and survival. Overall, tadpoles in the field performed much better than those in the common garden experiment, suggesting that animals either had an additional food source or some other factor promoted accelerated growth in the field enclosures. Results from both experiments are consistent with a plastic response by *R. catesbeiana*, as we found no evidence of origin (natal pond type) influencing the effects of *Phragmites*.

These findings imply that *Phragmites* provides substantially enhanced conditions for *R. catesbeiana* larvae in comparison with locally available native leaf litter. While our experiments do not offer a definitive explanation, they do provide clues. In the field experiment, improved larval performance was realized in spite of lower temperature and dissolved oxygen concentrations in *Phragmites* ponds. The common garden experiment was designed to isolate the role of *Phragmites* as a food resource. In common garden containers, the only food available was leaf detritus and the associated biofilm from either a *Phragmites* bed or the littoral edge of a control wetland. Water changes were reasonably frequent (every 3 to 5 days); at every change, new detritus was provided from wetlands and containers were washed. Our results suggest that *Phragmites* detritus provides superior food resources for *R. catesbeiana* tadpoles compared with the dominant native species contributing detritus in the study system, perhaps owing to litter properties such as lability or nutrient content. Interestingly, results from studies of invertebrates (amphipods, fiddler crabs,) also suggest a lack of harmful effects from a diet of *Phragmites* detritus [35]–[36].

While we were able to offer replication, our choice of control wetlands was limited by what existed in our local study region. Emergent vegetation other than *Phragmites* was uncommon in permanent ponds found in close proximity to our *Phragmites* sites; in most cases leaf litter from deciduous trees dominated the detritus along reference pond edges. As other researchers have noted, the origin (native versus nonnative) of litter of a plant is likely to be much less predictive of its influences compared to the relevant traits [18]. Other untested litter sources, including those of native origin, may equal or surpass *Phragmites* in qualities relevant to amphibian larval performance.

The positive effect of *Phragmites* on tadpole growth and survival was not a result of differences in consumption of tadpoles by predators between treatments. Both experiments were conducted in a way that largely eliminated the influence of predation on tadpoles. Predators were removed from detritus stocked into common garden containers. Post experiment searches of enclosure contents confirmed an absence of tadpole predators.

Our study does not address the overall influence of *Phragmites* on *R. catesbeiana* populations or on life history stages beyond early larval development. *Phragmites* tends to grow in dense thickets and its rapid growth can influence the hydroperiod of water bodies. Both of these effects could have a negative influence on breeding success of amphibians, which tend to lay their eggs along pond edges, and rely on ponds retaining water long enough for metamorphosis to be completed. However, dense stands of *Phragmites* could also provide superior cover from predators, both at the adult and larval stage.

In eastern North America, *R. catesbeiana* is a native species. In this context, available evidence provides no suggestion that nonnative *Phragmites* is a threat to *R. catesbeiana* populations. In western North America, interactions between the same two species have much different implications. The range of *R. catesbeiana* in regions where it is nonnative dwarfs its native distribution, with invasive populations established across much of the western US and parts of western Canada, Mexico, South and Central America, Hawaii, Japan, China, Korea and Europe [37]. Nonnative bullfrogs in the western US are seen as aggressive competitors and predators of native amphibians and are believed to be associated with the decline of some native populations [38]–[40]. Even if native species in the western US are not negatively influenced by invasive *Phragmites*, any positive effect of *Phragmites* on *R. catesbeiana* may enhance their impact on native amphibians. Indeed, Clarkson and DeVos [41] found a positive correlation between invasive *R. catesbeiana* and *Phragmites australis* presence in a riparian wetland environment in the lower Colorado River. If nonnative *Phragmites* were to gain a stronger foothold in the western US, the possibility of an ‘invasional meltdown’ [42], where facilitation by *Phragmites* could accelerate the negative impacts of nonnative *R. catesbeiana*, should be considered. Additional research into whether *Phragmites* supports a relatively more abundant or nutritious periphyton community, enhances native *R. catesbeiana* competitive dominance, or facilitates spread of nonnative *R. catesbeiana* remains a ripe area for future study.

*Phragmites* removal projects are justified in part on the assumed negative influence of *Phragmites australis* on wildlife [29]–[33]. However, evidence of negative impacts of *Phragmites* on wildlife appears to be taxon or life history stage specific [11], [43]–[46] and is tempered by reports of positive or no impacts on wildlife [11], [36]–[37], [43], [47]. In many cases evidence of impacts of *Phragmites* on native fauna is observational and may be limited to specific geographic areas [11], [48].

Why might *Phragmites* have positive effects on some native species and have neutral or negative impacts on others? The answer to this question would aid in predicting impacts of nonnative species on native species without testing these effects on a case-by-case basis. The results of this study and others [35]–[36] indicate that *Phragmites* provides nutritionally equivalent or even superior detritus compared with native sources (i.e. *Typha*, *Spartina*, deciduous leaf litter mix). Other characteristics of *Phragmites*, especially related to its tendency to alter hydrology, appear to lead to the loss of species, such as larval fish, that require higher flooding levels [44]. The thick decomposing litter beds associated with *Phragmites* can transform aquatic arthropod communities from plant to detritus based taxa [49]. The dense nature of *Phragmites* stands may serve as alternately a barrier to animal movement [11] or a source of cover [48].

Both empirical and observational studies can contribute to our understanding of the traits of an invasive species that lead to varying effects on native species, helping us to predict the potential impact on species of concern for management and conservation purposes.

Managers dealing with biological invasion intervene because some outcome is desired over alternatives. Desired outcomes are typically based on a combination of values and scientific information [50]–[51]. Just how values and information should be combined is currently the subject of debate. Some argue that values should influence the conduct of scientific research into biological invasions [52]. Others argue that studies of the impacts of nonnative species on native species, communities and ecosystems should be conducted while striving to avoid such biases [53]. The findings of this study suggest that study designs capable of detecting positive as well as negative effects of invasives may be particularly critical because successful management interventions require the mobilization of scarce resources. The flood of invasive species is growing faster than our efforts to deal with them. Approaches to efficient triaging of our management activities will rest on a firm foundation of documented benefits as well as harm.

## Materials and Methods

### Ethics Statement

The common garden and reciprocal transplant experiment methods were approved by the Yale University IACUC, under protocol number 2006–11040. Relevant permissions to conduct field work on privately owned property were obtained from individual land owners as well as the Branford Land Trust. The participation of residential landowners was contingent on guaranteeing their anonymity.

### Study Site Selection

We selected 10 wetlands in two cover categories (*Phragmites*, control) for study using a combination of remotely sensed and ground based information. *Phragmites* wetlands (<1.5 acres surface area) were identified by first locating potential sites using a 0.5 m resolution, false color infrared satellite image of the 22 coastal towns and cities between Milford and Old Lyme Connecticut, USA, a total area of 940 km^2^. An unsupervised classification led to a set of cover types potentially indicative of emergent wetland vegetation including *Phragmites*. This classified image was overlaid by U.S. Geological Survey National Wetlands Inventory coverage for the same area. We ground-truthed 182 wetlands (palustrine, ranging from seasonally flooded to permanently filled) including cover classes indicative of *Phragmites*, yielding 14 wetlands for which *Phragmities* dominated emergent vegetation and amphibians were present, and for which permission to work in the wetland was granted. Among these, we selected 5 to work in based on their high relative cover of nonnative *Phragmites* and the likelihood of finding sufficient amphibian embryos. Nonnative status of *Phragmites* stands was assessed using morphological criteria [54].

We identified control wetlands, those lacking *Phragmites*, by systematically searching permanent to seasonally flooded palustrine wetlands within 5 km of the selected *Phragmites* wetlands. In all, we investigated 108 potential control wetlands, yielding 27 wetlands for which *Phragmites* was absent, amphibians were present, and permission to use the wetland was granted. Among these, we selected 5 to work in based on their proximity to *Phragmites* wetlands, similarity in size and permanence, and the likelihood of finding sufficient amphibian embryos. We made every effort to include control matches with native emergent vegetation, but in most cases this type of littoral cover was absent. In one case a suitable control match could not be found within 5 km of the *Phragmites* wetland and the search radius was expanded to 15 km. The set of wetland pairs chosen for the study were located within a 450 km^2^ geographic area; all wetlands were between three and 30 km apart. The study area spanned from North Haven east to Westbrook along the coast of Connecticut, USA (west to east from approximately 72.82608 W, 41.41160 N to 72.46532 W, 41.31695 N).

### Embryo Collection

Once breeding activity began in early May, we checked each wetland for *R. catesbeiana* embryos every 1 to 4 days. Because *R. catesbeiana* eggs are indistinguishable from *Rana clamitans* (green frog) eggs, we identified hatchlings to species to ensure all larvae were *R. catesbeiana*. At least three egg masses were used to represent the *R. catesbeiana* population in each wetland in both experiments. If less than five egg masses were found, previously used masses were duplicated to add up to five replicates per wetland, per treatment (*Phragmites* and non-*Phragmites*) for an expected total of 100 replicates. In one *Phragmites* wetland only one egg mass was found; this wetland and its pair were not included in the analysis. Across the eight wetlands used in the study, we found a total of 31 egg masses, 14 from *Phragmites* ponds, and 17 from non-*Phragmites* sites. Details on the number of egg masses found and the frequency of duplication are included in Table S1. If five egg masses were found in a pond, we did not continue to search for additional egg masses, thus Table S1 represents the total number of egg masses found in each pond only if less than 5 masses were found.

### Field Experiment

A reciprocal transplant experiment was conducted in the field. Each *Phragmites* wetland was paired with a control wetland such that embryos were raised both in the natal and paired wetland of the opposing treatment.

Upon collection, equal sized egg mass portions were placed in floating 14 L clear plastic containers (Sterilite Inc, Townsend, MA, USA). Six 7 cm diameter holes were drilled into the container walls and covered with 0.78 mm nylon mesh to allow pond water circulation but prevent entry of predators. A 25×13 cm hole was cut in the lid and covered in 0.78 mm mesh to allow light penetration and prevent entry by predators. Foam was attached to the walls to allow floatation and air circulation within the container. Ten floating containers were attached to stakes hammered into the pond sediment along the littoral edge of each wetland at approximately 40 cm depth. In each wetland, five containers housed embryos in their natal pond and five contained embryos collected from the opposing treatment.

Screened enclosures were constructed to house tadpoles when they reached Gosner developmental stage 25 [55]. In each wetland, ten screened enclosures were staked in pairs in the pond sediment at approximately 50 cm depth, allowing tadpoles access to the surface, water column and sediment through a mesh barrier. Enclosures were constructed from columns of 122 cm tall vinyl coated garden fencing (GardenPlus, dist. by LG Sourcing, Inc., Wilkesboro, NC, USA), 50 cm in diameter, surrounded on all sides with 0.62 mm nylon mesh to allow water, algae, and small invertebrates to flow through the enclosures but exclude potential predators.

At least 7 days prior to stocking the enclosures with tadpoles, leaf detritus was collected from the littoral zone. Detritus was first dried for at least 6 days to eliminate potential aquatic insect predators. Approximately 11 L of dried, loosely packed detritus was placed in each enclosure for at least one day prior to stocking.

When tadpoles reached Gosner developmental stage 25, thirty individuals were selected haphazardly and transferred from the floating containers to a screened enclosure in the same wetland. An additional sample of thirty larvae were preserved in 70% EtOH and later measured for snout vent length and developmental stage. If an egg mass was used more than once, an additional thirty animals were selected for a second enclosure.

Enclosures were checked at least once each week during which time pH, dissolved oxygen, conductivity, and water temperature were measured near the center pair of enclosures at 10 cm depth (YSI 550A DO/Temperature meter, Oakton Instruments Waterproof pHTestr1 meter, Oakton Instruments Waterproof TDSTestr 3 meter). A HOBO logger attached to the outside of the center pair of enclosures recorded temperature measurements at 10 cm depth every 30 minutes until the final enclosure was removed from that wetland. If enclosure depth fell below 15 cm, all enclosures in that wetland were relocated to a depth of 40 cm. The experiment ended six weeks after stocking, at which point tadpoles were collected from enclosures, transported to the lab in pond water, wet weighed and then preserved in 70% EtOH. Snout vent length and developmental stage were measured after preservation.

To characterize species composition of the leaf detritus in the control and *Phragmites* wetlands, samples of detritus were collected from the littoral edge. Leaves were dried for two weeks, and one hundred leaves or leaf fragments were chosen haphazardly and identified to species wherever possible.

### Common Garden Experiment

Concurrent with the field experiment and using individuals from the same 31 egg masses originating from the eight study wetlands, a common garden experiment was conducted outside, on the grounds of the Marsh Botanical Gardens at Yale University. The experiment was designed to test the effect of origin (the type of pond where egg masses were laid – *Phragmites* or control) as well as treatment (*Phragmites* or control litter) and the interaction between origin and treatment. Five tables were set up with twenty 11.3 liter artificial containers (Rubbermaid, Fairlawn, OH, USA), arranged in a random block design on each table. In expectation of using five pairs of wetlands, one hundred containers were included in the experiment, with twenty containers allotted to each pair of wetlands. Two containers from each wetland (one *Phragmites* and one control treatment) were represented on each table, with sibships assigned to tables at random. Tables were set up beneath a shade cloth that allowed 50% light penetration.

Individuals from each egg mass were kept in separate containers for the duration of the experiment. Equal sized portions of egg masses were placed into containers filled with dechlorinated tap water, aged three or more days. After 9–13 days, when the tadpoles had reached Gosner stage 25, the experiment was stocked. We haphazardly selected twelve tadpoles from each egg mass to be allocated to a control treatment and an additional twelve animals for the *Phragmites* treatment. A sample of twelve tadpoles was weighed and then preserved as a record of the initial size. If an egg mass was duplicated (in the instance of finding three or four egg masses in a wetland), an additional 24 tadpoles were selected – twelve for each treatment.

Tadpoles were stocked into dechlorinated, aged tap water and were provided with detritus as the only food source. A mixture of detritus collected from two control wetlands and from two *Phragmites* wetlands was used throughout the experiment. Litter collected from the control wetlands consisted of a mixture of deciduous tree leaves, while litter collected from the *Phragmites* wetlands consisted of 100% *Phragmites* leaf detritus. To avoid water fouling by the tadpoles, every three to five days thereafter water and leaves were replaced after containers had been scrubbed. Throughout the experiment, fresh litter was collected within five days of feeding. Leaves remained submerged in pond water until use. At every water change, each container received a 15×15 cm single layer of leaf detritus. Snails and potential predators such as beetle larvae or dragonfly larvae were removed from the leaves before feeding.

Containers were taken down six weeks after they were stocked; tadpoles were then wet weighed, preserved in 70% EtOH and later measured (SVL) and staged. Since egg masses were collected over a period of two months, some sibships reached this 6 week mark earlier than others.

Seven Hobo loggers haphazardly spread across the five tables recorded water temperature throughout the experiment. Dissolved oxygen, pH and temperature were measured across stocked containers four times on the day before a water change (using a YSI 550A DO/Temperature meter and an Oakton Instruments Waterproof pHTestr1).

### Statistical Analysis

To evaluate effects of *Phragmites* on *R. catesbeiana* larval performance, separate Multivariate Analyses of Variance were conducted for the field and common garden experiments. Response variables included final mass, final developmental stage and survival rate with common garden container or field enclosure serving as the unit of measure. Mass and stage were averaged among survivors within an enclosure or container. Effects of Origin (whether the egg mass was collected from a *Phragmites* or control wetland), Treatment (*Phragmites* or control) and the interaction between Origin and Treatment were evaluated. Final mass and developmental stage were log transformed and survival rate was arcsine square root transformed to meet MANOVA assumptions of equal variance among groups and multivariate normality of deviations from the group means. Where significant multivariate effects were uncovered, univariate ANOVA tests were carried out. A significant interaction between Origin and Treatment effects in the multivariate or any univariate analysis would suggest evidence of divergence among populations in response to exposure to *Phragmites* in the natal environment. Analyses were conducted using the SAS Proc GLM function (SAS Statistical Software 9.1.3). Out of 80 enclosures in the eight wetlands used in the analysis, six field enclosures were discovered with torn mesh. Data from these enclosures and the corresponding replicate in the reciprocal transplant was excluded from analysis.

To test for any differences in water chemistry variables owing to treatment, a multivariate analysis of variance was conducted for each experiment. In the field, dissolved oxygen, conductivity and pH were included as response variables. For the table experiment, response variables included temperature, pH and dissolved oxygen. Univariate ANOVA tests were then carried out if the overall model was significant. For the field experiment, temperature was analyzed by averaging the temperature over the period experienced by tadpoles in each enclosure and conducting a univariate ANOVA comparing temperatures experienced in the *Phragmites* and non-*Phragmites* wetlands. For this parameter the enclosure was the unit of measure, while for the other field water chemistry measures the pond was the unit of measure. Temperature in the common garden experiment was measured in each container along with the other water chemistry parameters, thus temperature was included in the MANOVA for this experiment. Water chemistry analyses were conducted using the SAS Proc GLM function.

## Supporting Information

Table S1 **Number of egg masses included in the two experiments and duplication frequency across the study ponds.** Pond numbers refer to pairing, C and P refer to non-*Phragmites* (control) and *Phragmites* sites and correspond with pond names in Table 4.
(DOC)
