# Supplementary material for: Positive Effects of Nonnative Invasive Phragmites australis on Larval Bullfrogs
Source: PLoS One. 2012 Aug 30;7(8):e44420. doi: 10.1371/journal.pone.0044420 (PMC3431391; doi:10.1371/journal.pone.0044420)
Supplement: Table S1 — Number of egg masses included in the two experiments and duplication frequency across the study ponds. Pond numbers refer to pairing, C and P refer to non-Phragmites (control) and Phragmites sites and correspond with pond names in Table 4. (DOC) [file pone.0044420.s001.doc]

Appendix S1

Table S1. Number of egg masses included in the two experiments and duplication frequency across the study ponds. Pond numbers refer to pairing, C and P refer to non-*Phragmites* (control) and *Phragmites* sites and correspond with pond names in Table 4.

| Pond Name | Egg masses | Egg masses duplicated |
| --- | --- | --- |
| C-1 | 5 | 0 |
| P-1 | 3 | 2 |
| C-2 | 4 | 1 |
| P-2 | 5 | 0 |
| C-3 | 4 | 1 |
| P-3 | 3 | 2 |
| C-4 | 4 | 1 |
| P-4 | 3 | 2 |
| C-5^a^ | 4 | N/A |
| P-5 ^a^ | 1 | N/A |

^a^ This pair was excluded from the analyses, as only one egg mass was found in P-5.
